# High-Throughput Diagnostic Assay for a Highly Prevalent Cardiomyopathy-Associated *MYBPC3* Variant

**DOI:** 10.4172/2155-9929.1000303

**Published:** 2016-09-30

**Authors:** David Y Barefield, Thomas L Lynch, Aravindakshan Jagadeesan, Thriveni Sanagala, Sakthivel Sadayappan

**Affiliations:** 1 Department of Cell and Molecular Physiology, Loyola University, Chicago, USA; 2 Department of Cardiology and Echocardiography and Cardiographics, Loyola University, Chicago, USA; 3 Center for Genetic Medicine, Feinberg School of Medicine, Northwestern University, Chicago, IL, USA

**Keywords:** Hypertrophic cardiomyopathy, Genotype-phenotype, South Asian population, RNaseH qPCR, DNA diagnostic test

## Abstract

A 25-basepair deletion variant of *MYBPC3* occurs at high frequency in individuals of South Asian descent and is estimated to affect 55 million people worldwide, carrying an increased likelihood of cardiomyopathy. Since this variant is prevalent and severe in this subpopulation, quick and affordable screening to provide risk-assessment to guide treatment for these patients is critical. An RNaseH qPCR assay was developed to quickly and specifically diagnose the presence of the 25-basepair deletion variant in *MYBPC3*. RNAseH-blocked nucleotide primers were designed to identify the presence or absence of the wild type *MYBPC3* allele or the genomic sequence containing the 25-basepair deletion. Using this assay, three blinded operators were able to accurately determine the genotype from human genomic DNA samples from blood and saliva using a qPCR thermocycler. Furthermore, positive variant subjects were examined by both electrocardiography and echocardiography for the presence of cardiomyopathy. A simple, robust assay was established, verified and validated that can be automated to detect the presence of the highly prevalent 25-basepair deletion *MYBPC3* variant using both blood and saliva samples. The assay will provide quick and accurate prescreening of individuals at high risk for cardiomyopathies and allow for better clinical identification of 25-basepair deletion *MYBPC3* carriers in large cohort epidemiological studies.

## Introduction

Hypertrophic cardiomyopathy (HCM) is a global genetic heart disease affecting at least 1 in 500 people, including approximately 600,000 people in the United States and 14.25 million people worldwide [1,2]. HCM is typically characterized by excessive left ventricular hypertrophy to the point of causing left ventricular outflow obstruction, diastolic dysfunction, myocardial ischemia, and mitral regurgitation [3,4]. Importantly, HCM is also a commonly identified cause of sudden cardiac death in young people and athletes [2,5–8]. It is predominantly caused by mutations in genes encoding proteins of the sarcomere-the basic unit of striated muscle tissue-including one of the two most common HCM-associated genes, *MYBPC3* [9,10]. Since 1995, nearly 300 disease-causing variants have been published for *MYBPC3*, the gene that encodes cardiac myosin binding Protein-C (cMyBP-C) [11,12]. cMyBP-C is a key thick filament protein, connecting both myosin and actin filaments, and contributes to the regulation of sarcomere structure and function. Even though mutations in *MYBPC3* account for approximately 42% of all HCM cases, its genotype-phenotype relationships remain poorly understood [12]. Therefore, genetic screening and clinical follow-up for carriers of common cardiomyopathy-associated sarcomere mutations would shed light on the pathogenic mechanisms of HCM and management of the disease [13,14].

Previously, we discovered a 25-basepair (bp) deletion in the *MYBPC3* gene, identified as *MYBPC3*^ΔInt32^, to be particularly prevalent among individuals of South Asian ancestry [15,16]. This variant is the focus of the current study and is characterized by the loss of the splicing branch point in intron 32, leading to the skipping of exon 33 and a frameshift starting in exon 34, This results in the replacement of 62 amino acids with a novel 55 amino acid sequence in the C10 domain of the C-terminus of cMyBP-C. It is estimated that 55 million people worldwide carry this variant, which is associated with the development of HCM and heart failure (HF) [14]. Importantly, heterozygous *MYBPC3*^ΔInt32^ variant carriers have highly variable disease onset and typically develop symptoms of HCM during the third decade of life. Strikingly, an additive effect of *MYBPC3* mutations with other sarcomeric protein mutations, a so-called “two-hit phenomenon,” results in severe cardiac hypertrophy, early sudden cardiac death [17–19] and coronary artery disease [20,21]. For example, if carriers of the *MYBPC3*^ΔInt32^ variant also carry a mutation in β -myosin heavy chain, this combination frequently results in sudden cardiac death [22]. Homozygous carriers of this *MYBPC3*^ΔInt32^ variant develop severe cardiomyopathy, typically in the second decade of life, with dilation of the ventricle and often HF [23]. The high frequency of this variant in South Asian populations has been established in studies in South Asia, but the distribution of the variant in South Asian populations living in the USA and elsewhere remains to be studied [23]. Individuals of South Asian descent in the USA number about 3 million. Because of the potential severity and high frequency of this variant, rapid and effective detection is critical to allow for timely patient counseling. Based on initial studies, we estimate that 11.22% of the South Asians living in the USA carry the *MYBPC*^ΔInt32^ variant [24], which is a significantly higher number than had been expected [23].

Despite these statistics, a standard genetic test for clinical diagnosis has never been established for screening populations at high risk for cardiomyopathies [25]. Given these alarming statistics, as well as the further increased incidence of this mutation among South Asians compared to Caucasians, this pilot study is aimed to establish a sensitive, variant-specific qPCR assay to efficiently determine an individual’s genotype by genomic DNA collected from a small blood or saliva sample.

This assay is best understood in the context of global research efforts to develop genotype-phenotype relationships as a framework for understanding the pathological potential of specific variants allowing for more accurate assessment of individual disease risk in a clinical setting [26,27]. Moreover, adding this test to the existing diagnostic tool kit will allow the frequency and severity of this variant to be determined over time by studying clinical outcomes [28]. Importantly, since this *MYBPC3*^ΔInt32^ variant is highly prevalent, large-scale clinical screening can determine if this variant is a direct cause of cardiomyopathies or is a risk allele that acts with other pathogenic variants, or environmental factors. In either case, future findings would prove valuable for understanding disease progression in affected individuals and populations.

## Materials and Methods

Collection of human samples-collection of human blood and saliva samples and the experiments used to examine them were approved by the institutional review board at Loyola University Chicago (LU# 205109 and LU# 207359). This study conformed to the principles outlined in the declaration of Helsinki. Subjects were recruited from the United States and were primarily individuals of South Asian descent. All subject samples were deidentified before use in these experiments. Subjects who were carriers were then identified and single-blindedly contacted for a voluntary follow-up echocardiogram and electrocardiogram, after IRB approval (LU #207377).

DNA isolation and traditional genotyping-genomic DNA was isolated from either whole blood samples taken by venipuncture or saliva samples in accordance with the approved IRB for this study. Blood and saliva samples were processed using the QIAGEN QIAamp® DNA Mini Kit (Catalog Number 51104) to obtain genomic DNA with final concentrations approximately 250 nM. This template was used for traditional PCR using two primers (Forward: 5’-GTT TCC AGC CTT GGG CAT AGT-3’; Reverse: 5’-GAG GAC AAC GGA GCA AAG CCC −3’) in the following reaction as described previously 29. REDTaq® ReadyMix™ PCR reaction mix (20 mM Tris-HCl, pH 8.3, with 100 mM KCl, 3 mM MgCl_2_, 0.002% gelatin, 0.4 nM dNTP mix, stabilizers, and 0.06 unit/ul of Taq DNA Polymerase) was diluted 2x in nuclease-free water, and 25 pmol each of forward and reverse primer and 50 ng of DNA were added to the mix. PCR reactions were run using the MyCycler™ thermal cycler (BioRad) with the following conditions: 94°C (30 sec); Denaturation, 60°C (45 sec); Annealing (72°C and 30 sec), and 72°C extension (1 min), followed by a final extension at 72°C for 10 minutes. PCR samples were resolved on a 2.5% agarose gel and visualized with ethidium bromide. The *MYBPC3*^ΔInt32^ variant was easily identifiable using this method, with a 403 bp WT product and a 378 bp mutant ^ΔInt32^ product [29].

### Electrocardiogram

A resting 12-lead ECG was recorded prior to echocardiography acquisition to determine the presence of left ventricular hypertrophy and arrhythmias as described and recommended by the 2014 European Society of Cardiology guidelines [5,30].

### Echocardiography

Echocardiography was performed to assess cardiac structure and function. Acquisition and analysis was performed using a GE Vivid 7 and 9 ultrasound system with an M4S probe at Loyola University Medical Center. Left ventricular posterior wall (LVPW) thickness, interventricular septal (IVS) thickness, left ventricular systolic diameter (LVSD), and left ventricular diastolic diameter (LVDD) were measured in parasternal long-axis view, as recommended by 2015 American Society of Echocardiography and 2014 European Society of Cardiology guidelines [5,31]. The Teichholz method was used to calculate left ventricular ejection fraction. Mitral inflow pulsed wave Doppler was used to measure early diastolic mitral inflow (E value) velocity and late diastolic mitral inflow (A wave) velocity. Tissue Doppler was used to measure the early diastolic relaxation velocity of mitral annulus at the septum (septal E’) and at the lateral wall (lateral E’). Septal E’ was used to calculate E/E’ ratio [32,33].

### Quantitative PCR genotyping

A qPCR assay was used for genotyping by amplifying human plasmid DNA constructs or human genomic DNA on a BioRad CFX96 qPCR thermocycler with the SybrGreen method of amplification quantification. Analysis was performed using BioRad’s CFX software. Samples were run in triplicate using 20 µl reaction volumes with 1 ng to 10 ng genomic DNA or 1 pg to 10 pg plasmid DNA, with 25 nM working primer concentration and an RNaseH enzyme concentration of 200 mU/uL (Integrated DNA Technologies, Inc., Coralville, Iowa) [34].

The reaction was run at 95°C for 3 minutes, followed by 50 cycles of 95°C for 15 seconds, and 60°C for 30 seconds, followed by reading of SybrGreen fluorescence. Effectiveness of the assay requires clean PCR reagents and RNAseH enzyme that is working properly, making proper reagent quality control necessary.

### Statistical methods

For these assays, ΔCq=Cq^(WT)−^Cq^(ΔInt32)^. Comparisons of ΔCq values between WT, Het and ΔInt32 samples were made using a oneway ANOVA with Tukey’s multiple comparisons post-test. Conversion of ΔCq values into fold change values was calculated as fold change=2^ΔCq^. Significance was set as p<0.05. Values are reported as mean ± standard error of the mean, unless otherwise specified. Receiver operator characteristic (ROC) curves were calculated with a 99% confidence interval. Statistics were performed using GraphPad Prism 6.

## Results

**Figure 1:**
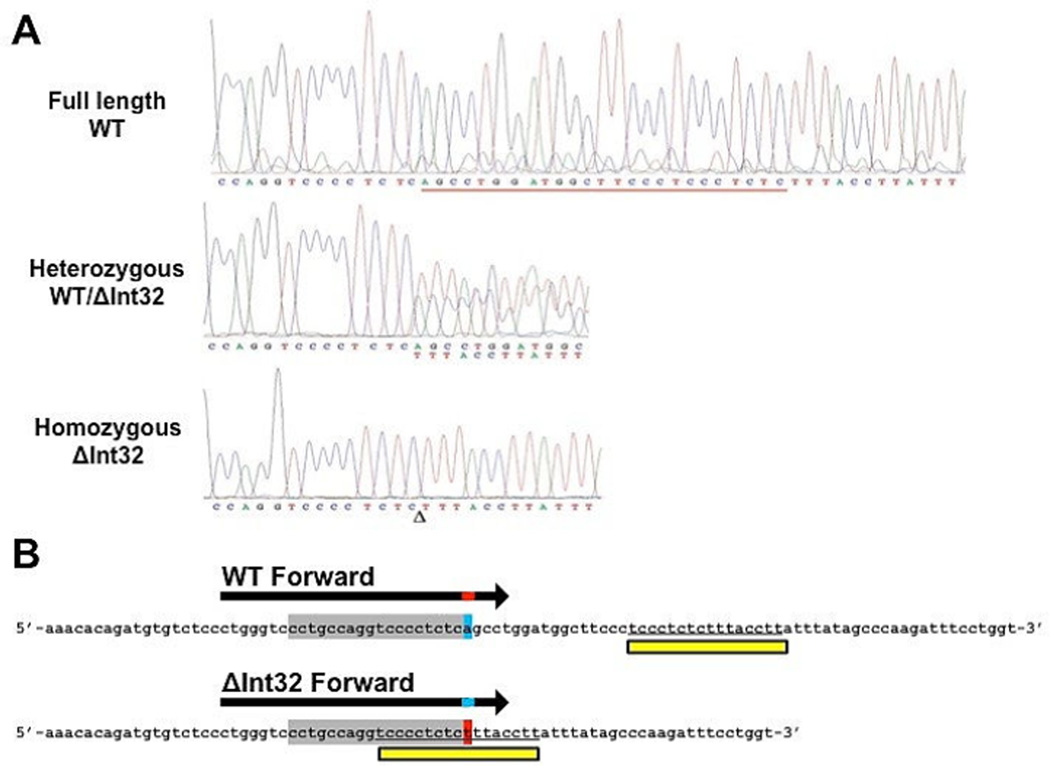
Development of a specific assay to recognize the *MYBPC3*^ΔInt32^ variant. (a) Sequencing chromatograms of human genomic DNA taken from an individual not carrying the ΔInt32 variant (top), heterozygous for the ΔInt32 variant (middle), and homozygous for the variant (bottom). (b) Schematic of the RNaseH primers used to identify the presence of the 25 bp deletion in the *MYBPC3*^ΔInt32^ gene. WT and ΔInt32 forward primers shared the same primary sequence of deoxynucleotides (grey nucleotides), with a single mismatched ribonucleotide base (red/blue nucleotide). Cleavage of only mismatched ribonucleotides allows the primer to amplify the sequence. The mutant sequence created by the removal of the 25 bp is repeated exactly in the WT sequence 3’ to the site of deletion, preventing the use of primers that span the splice site for detection of the *MYBPC*^ΔInt32^ variant (yellow boxes).

Real-time qPCR to determine the presence of the *MYBPC3*^ΔInt32^ variant-this *MYBPC3*^ΔInt32^ variant presents several challenges for designing specific qPCR assays, as detailed in the discussion section and Figure 1.

As a result, traditional two-primer approaches, splice-site spanning Taq Man systems, and LNA primers were all initially tried without success (data not shown). Therefore, taking a novel approach, we used a primer design that incorporates a modified nucleotide blocking strategy. Specifically, we employed a blocked-primer strategy using RNaseH cleavage of a specific mismatched ribonucleotide base in a GEN2 RNaseH primer that removes a 3’ blocking sequence, allowing primer extension (Integrated DNA Technologies, Inc. Coralville, Iowa) [34]. This technology effectively prefers amplification of the appropriate sequence by only allowing primer amplification in the presence of specifically cleaved mismatched ribonucleotide base. Previously, this approach worked well to detect single nucleotide polymorphisms (SNPs) and other elusive sequences [34]. For this assay, we used a forward primer specific for the wild-type (WT) allele designed with one ribonucleotide (underlined) base mismatched to the WT sequence followed by non-extendable blocking bases (5’-CCT GCC AGG TCC CCT CTC rAG/iSpC3//iSpC3/T C-3’). This primer will only function if the ribonucleotide mismatches to the WT template, allowing cleavage and extension of the primer. A second primer was used with specificity for the *MYBPC3*^ΔInt32^ mutant allele (ΔInt32) that contains a ribonucleotide base mismatched to the mutant sequence (5’-CCT GCC AGG TCC CCT CTC rUT/iSpC3//iSpC3/C G-3’). In this nomenclature, iSpC3 refers to IDT’s spacer sequence. We also used a common reverse primer (5’-AGA GTC AAC ACT CCC TGC T-3’) using standard DNA bases that recognize both WT and ΔInt32 sequences. These two specific ribonucleotide-mismatch forward primers and one universal reverse primer allow cleavage of the genotype-appropriate primer and preferential amplification of the corresponding allele (Figure 1b).

### The RNaseH qPCR assay shows specificity using plasmid DNA sequences

In order to test the functionality of these RNaseH qPCR primer sets, initial testing was performed using plasmid DNA containing the genomic sequence of the *MYBPC3*^ΔInt32^ variant and the human genomic wild-type sequence, as shown in Figure 1a. With the two allele-specific mismatched forward primers, it is expected that the WT sequence will show more robust amplification and signal when amplified by the WT assay, whereas the *MYBPC3*^ΔInt32^ variant will be preferentially amplified by the mutant-specific primer. In addition, it is expected that an equal mixture of template should yield an equal level of amplification using both primers.

**Figure 2:**
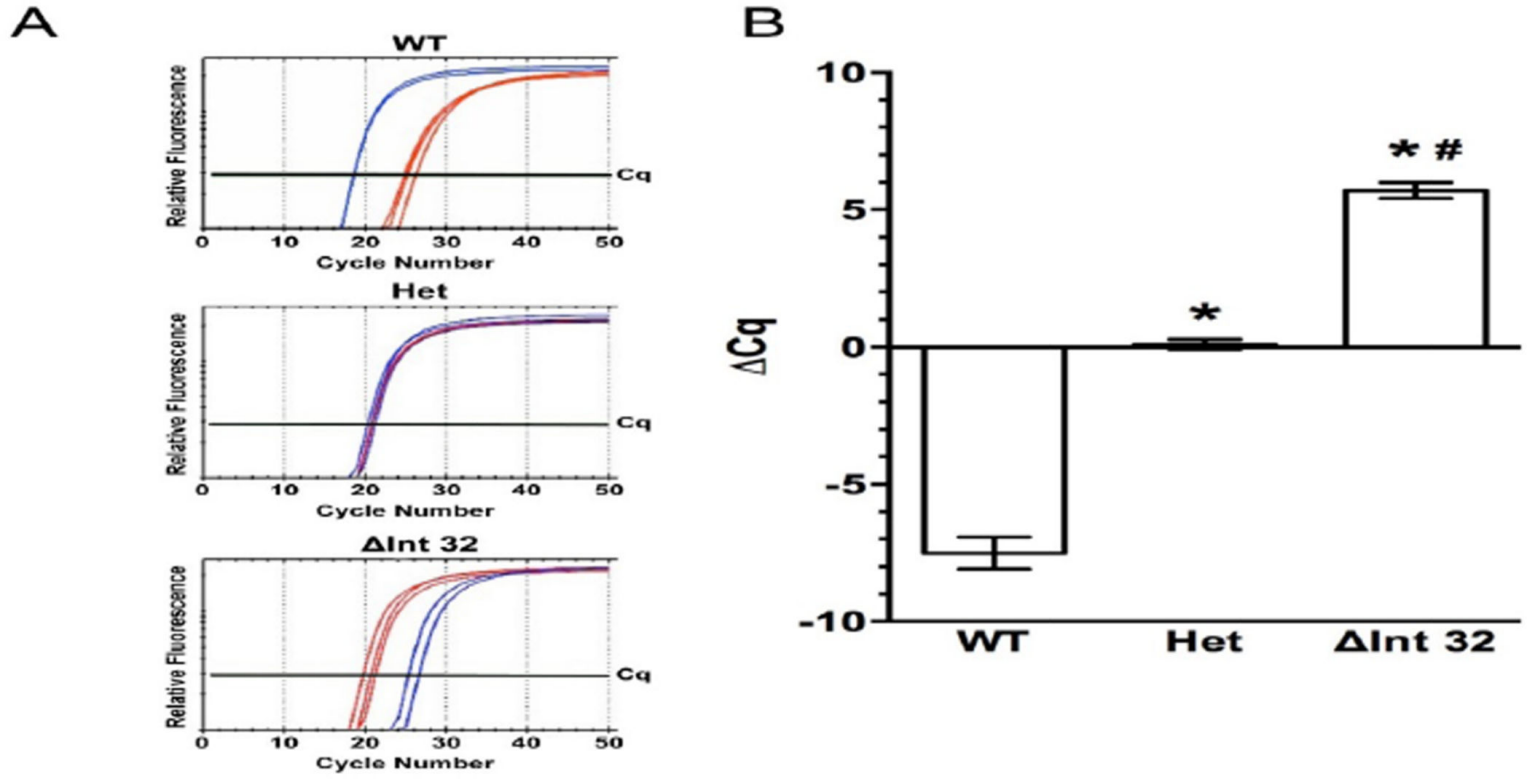
Determining specificity and selectivity of the diagnostic assay using plasmid constructs of the mutation. (a) Representative SybrGreen amplification curves of WT (blue traces) and ΔInt32 (red traces) RNAseH qPCR primers on plasmid containing the WT genomic *MYBPC3* sequence (top), a 1:1 ratio of WT and ΔInt32 genomic DNA sequence “Het” (middle) and homozygous *MYBPC3*^ΔInt32^ (bottom). (b) ΔCq values for WT, Het, and homozygous *MYBPC*^ΔInt32^ using the WT-and ΔInt32-specific RNAseH assay. ΔCq=Cq(WT)-Cq(ΔInt32). n=3 for all genotypes. All values shown as mean ± SEM. *=P<0.001 vs. WT. #=P<0.001 vs Het.

Amplification of the WT plasmid showed that the WT probe reached the quantitation threshold 7.5 ± 1.0 cycles ahead of the *MYBPC3*^ΔInt32^ variant (ΔInt32) probe, indicating a 1 ×102. 27-fold preference of amplification in the WT assay compared to the mutant assay using WT template (Figure 2a and 2b). A 1:1 mixture of WT and ΔInt32 plasmid was used to model a heterozygous condition, which resulted in a difference of 0.1 ± 0.3 cycles between the cycle of quantification (Cq) values for WT and ΔInt32 products. This showed that both assays work at equal efficiency when both templates are present. The ΔInt32 probe amplification of the ΔInt32 plasmid reached the quantitation threshold 5.7 ± 0.5 cycles before the WT probe, showing a 1 × 101.72 fold increase in amplification of the mutant assay compared to the WT assay using the mutant template. In these cases, low-level amplification was observed from the WT probe with ΔInt32 plasmid and from the ΔInt32 probe with WT plasmid. This factor is essential in confirming that the assay reaction did not fail. If the WT or ΔInt32 assay did not normally show low-level amplification of the opposite template, a lack of amplification from one assay could incorrectly report the absence of the target allele, resulting in an incorrectly identified genotype, when in fact the reaction has simply failed to occur, leading to false negative results. These results indicate that both WT and ΔInt32 assays prefer the correct primer/probe combination by several orders of magnitude using plasmid templates. These factors allow ΔCq (difference between WT and ΔInt32 assay Cq) values to be calculated in order to determine the correct genotype (Figure 2b).

### The RNaseH qPCR assay can determine genotype from human genomic DNA

**Figure 3:**
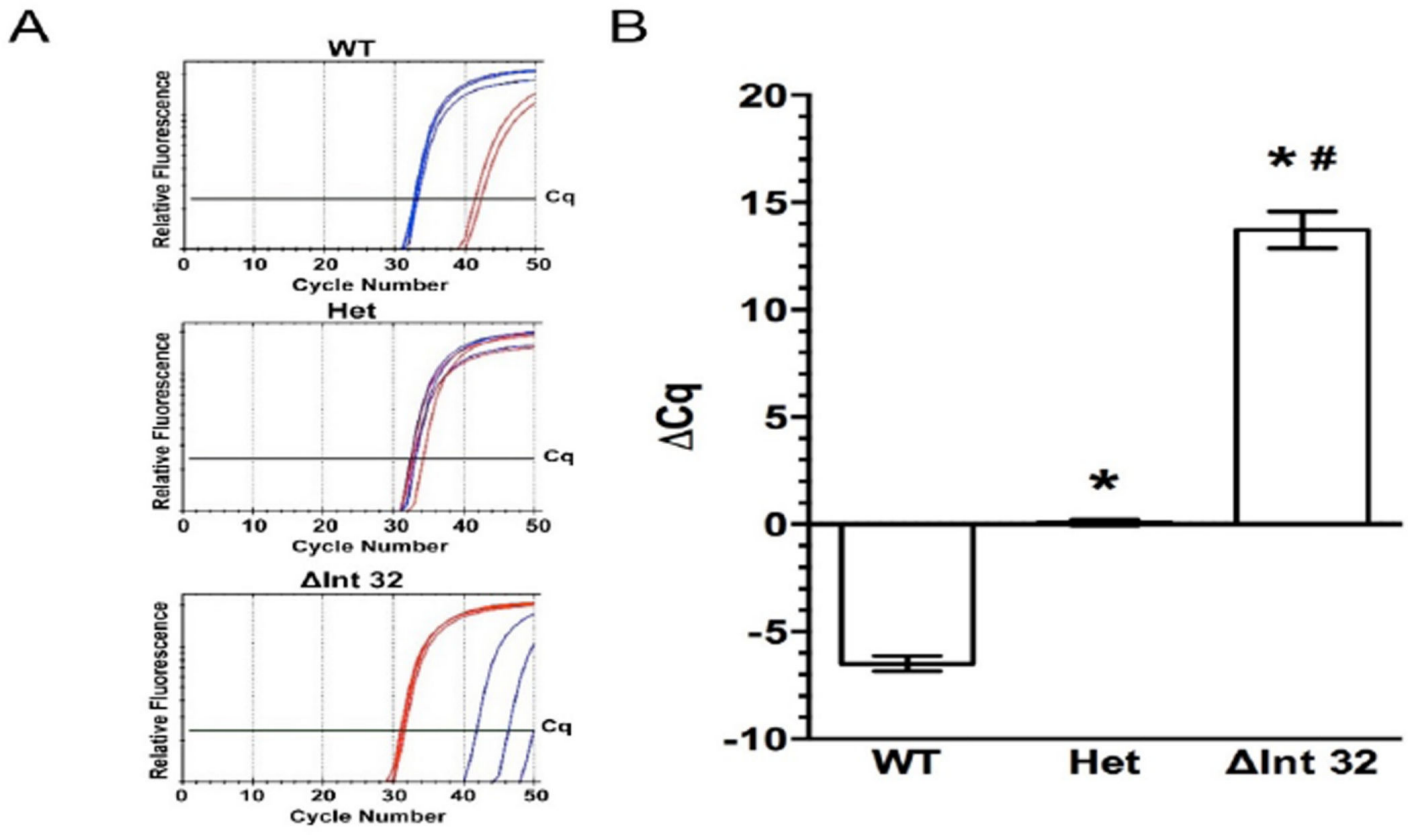
Establishing the diagnostic assay using human genomic DNA samples, (a) Representative SybrGreen amplification curves of WT (blue traces) and ΔInt32 (red traces) RNAseH qPCR primers on human genomic DNA of known genotype from WT (top), Het (middle) and homozygous *MYBPC3*ΔInt32 (bottom). (b) ΔCq values for WT (n=13), Het (n=9), and homozygous *MYBPC3*ΔInt32 (n=4) using the WT-and ΔInt32-specific RNAseH assay. ΔCq= Cq^(WT)^-Cq^(ΔInt32)^. All values shown as mean ± SEM. *=P<0.001 vs. WT. #=P<0.001 vs Het.

**Figure 4:**
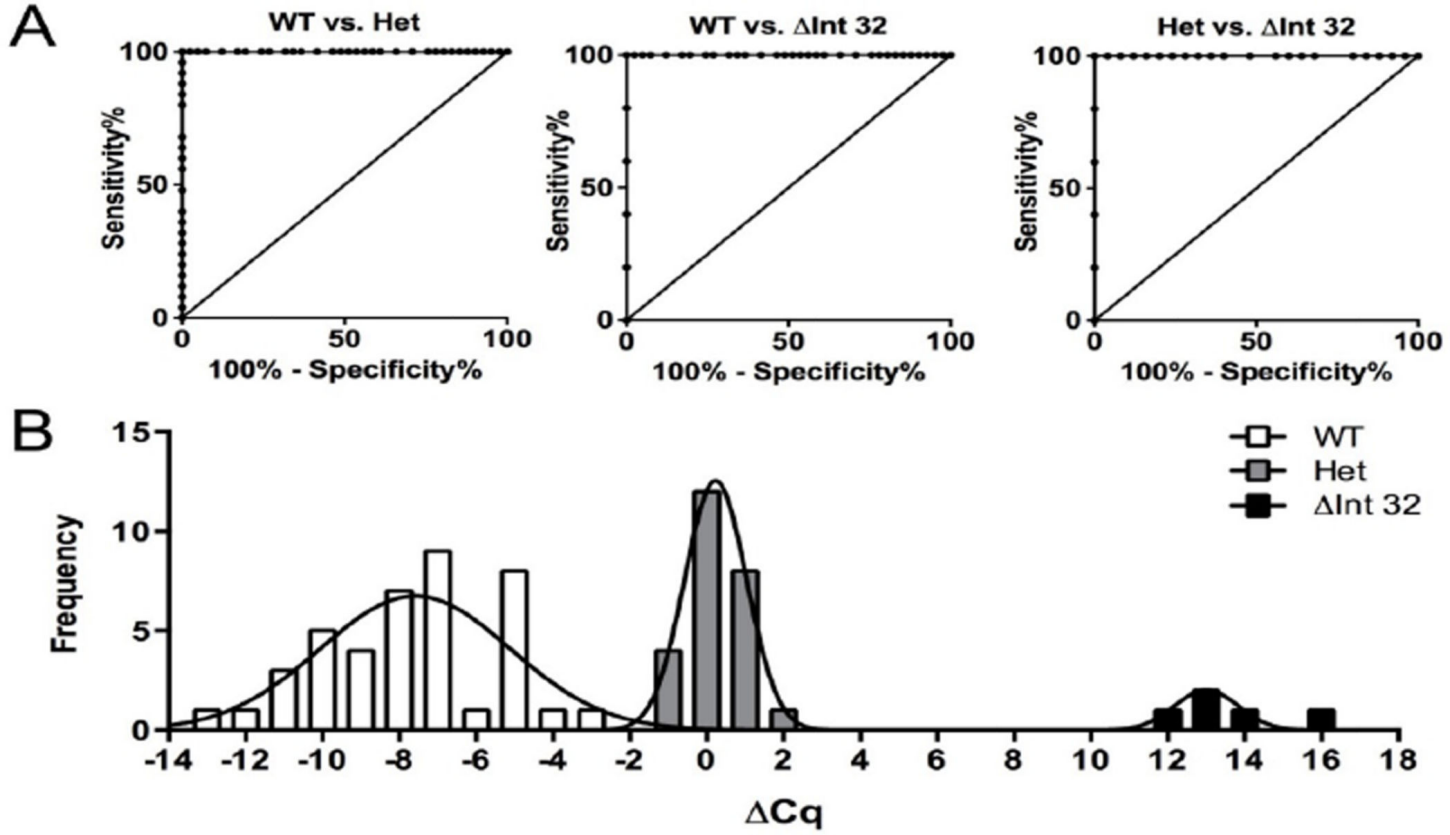
Assessment of the sensitivity and specificity of the ΔInt32 assay. (a) ROC curves showing perfect sensitivity and specificity for genotype calls based on ΔCq values between WT and Het, WT and ΔInt32, and Het and ΔInt32 from assay results using human DNA samples. ROC curves were calculated using 99% confidence intervals. (b) Histogram with Gaussian fit curves illustrating the separation of the ΔCq values between the three genotypes. WT n=41, Het n=25, ΔInt32 n=5.

After confirming the functionality of the primers using plasmid DNA, we validated this assay using human genomic DNA isolated from blood samples previously genotyped by traditional PCR. In mutation-negative samples, the WT primer reached the threshold 6.48 ± 1.25 cycles before the ΔInt32 primer (Figure 3a and 3b), indicating approximately an 1 × 10^2^ fold preference for the WT assay, similar to the results observed using WT plasmid template. In samples known to be homozygous for the *MYBPC3*^ΔInt32^ variant, the ΔInt32 primer outperformed the WT primer with a ΔCq of 13.72 ± 1.70 cycles. This preferential amplification of the ΔInt32 assay (a magnitude of 1 × 10^4.16^) compared to the WT assay when using homozygous *MYBPC3*ΔInt32 genomic DNA was even greater than the preferential amplification using plasmid samples, as shown above. Importantly, the performance of both WT and ΔInt32 assays were comparable when using known heterozygous genomic DNA samples, with a ΔCq of 0.08 ± 0.45. These results also reflect the results using plasmid template. The RNAseH qPCR assay shows high sensitivity and specificity of detection between genotypes. The ΔCq values calculated from the results of the WT and ΔInt32 assays using WT, heterozygous, and homozygous genomic DNA were used to generate ROC curves to establish the sensitivity and specificity of the assay (Figure 4a). These curves showed 100% sensitivity and specificity for differentiating WT from Het, WT from ΔInt32, and Het from ΔInt32.

The accuracy of this test can be seen in the clear separation of ΔCq values for all three genotypes (Figure 4b). Using this information, we set the ΔCq cutoffs for genotype calls between −14 and −2 for WT, −2 and 2 for Het, and 8 and 19 for ΔInt32. These values were used for genotyping unknown samples. The RNaseH qPCR assay can be used to effectively genotype the *MYBPC3*^ΔInt32^ variant in human DNA samples-We next used this assay on unknown human genomic DNA isolated from blood samples to confirm that it would allow a blinded operator to perform accurate genotyping. These samples were all run along with genotype-positive controls and no template controls (NTC) for comparison. The unknown samples were genotyped using the ΔCq of the amplification curves between the WT and ΔInt32 probes and the cutoffs set from Figure 4.

**Figure 5:**
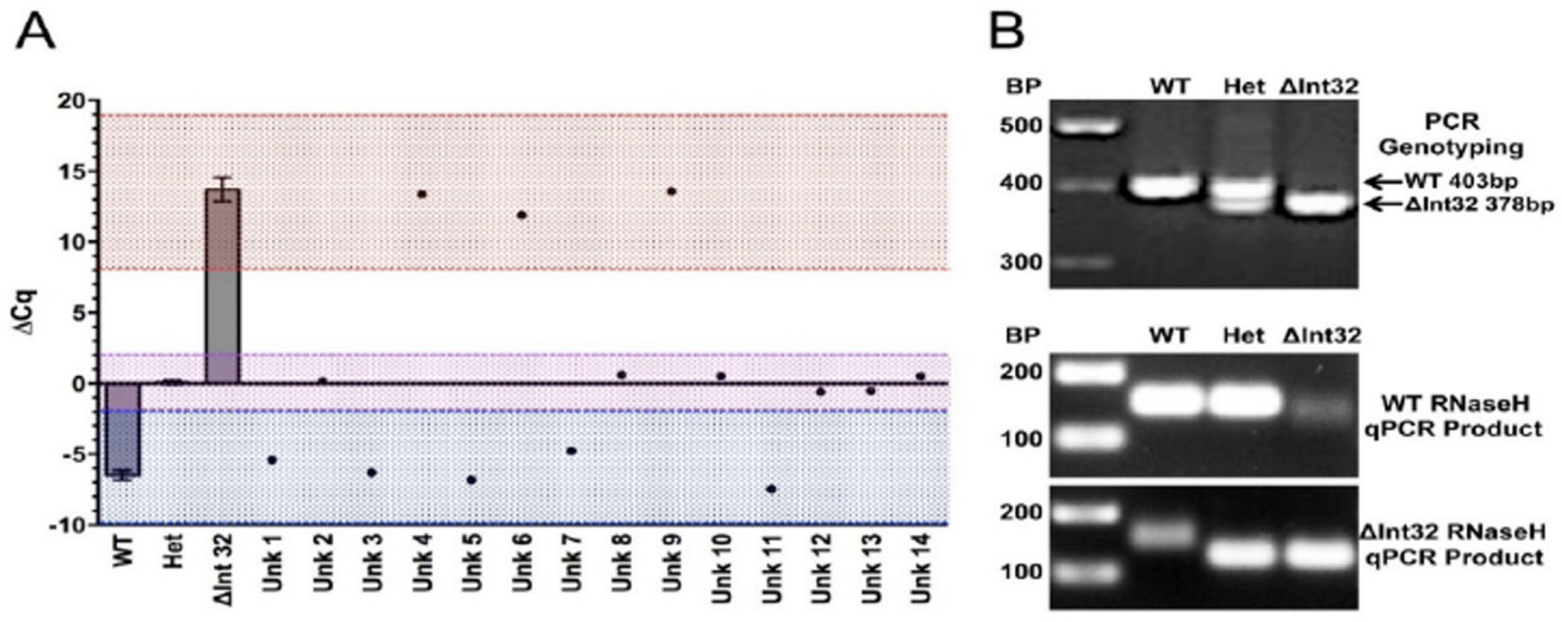
ΔCq values are used to blindly report genotype of unknown samples. (a) ΔCq values of 14 unknown samples compared with the average of known WT, Het, and homozygous *MYBPC3*ΔInt32 samples from Figure 3. Dashed lines indicate the cutoffs used to call genotypes for WT (blue), Het (purple), and homozygous *MYBPC3*ΔInt32 (red). Genotype calls were made if the ΔCq values for unknown samples fell between −14 and −2 for WT, −2 and 2 for Het, and 8 and 19 for homozygous *MYBPC3*ΔInt32. (b) The genotype of unknown samples called using the RNAseH assay was confirmed with traditional PCR and resolved on agarose gels. (c) Following RNaseH qPCR the amplified products were resolved on agarose gels showing the expected products for the respective assays.

The genotypes of unknown samples were called by three blinded operators using the ΔCq values generated from the RNAseH qPCR assay (Figure 5a). It was decided a priori that any unknown samples not falling within any of those Cq ranges would be considered failed and rerun, although this did not actually occur for any unknowns. The accuracy of genotype calls using the RNaseH qPCR assay was confirmed by traditional PCR and agarose gel electrophoresis (Figure 5b), with all calls from three observers correct and in agreement (Table 1). Gel electrophoresis of the RNaseH qPCR product showed the major WT and ΔInt32 products with a 25 bp size difference, as expected (Figure 5c). A minor product was detected by agarose gel from the WT assay using a ΔInt32 sample and the ΔInt32 assay using a WT sample. These products correspond to minor amplification of the opposite target observed using both plasmid DNA and known human DNA with and without the mutation (Figures 2 and 3).

Assay validation using DNA extracted from saliva samples. The RNaseH qPCR assay was next used with DNA samples isolated from saliva samples. Saliva collection is simple, noninvasive, does not require a phlebotomist, and facilitates the transport and isolation of DNA. Samples were collected from 12 South Asian subjects, and 200 µl of saliva were used to extract DNA for genotyping. The RNaseH qPCR assay was performed as described in Methods and blindly verified with traditional PCR. Two out of 12 samples were positive for the presence of the *MYBPC3*^ΔInt32^ variant. In both instances, these were heterozygous individuals (Table 2). Based on the results from these two experiments, it can be concluded that the RNaseH qPCR assay is accurate and can be readily performed to determine the presence of the *MYBPC3*^ΔInt32^ variant regardless of the source of genomic DNA, provided the DNA is isolated properly and is of good quality.

### Echocardiographic analysis of heterozygous carriers and non-carriers of the *MYBPC3*^ΔInt32^ variant

We previously determined that *MYBPC*^ΔInt32^ is linked to the development of cardiomyopathies [23]. However, to determine if it is associated with the development of HCM, echocardiography and ECG recordings were performed in a selected group of individuals of South Asian descent living in the USA who had also consented to our diagnostic genotyping assay. The subject group consisted of nine heterozygous *MYBPC*^ΔInt32^ variant carriers. Clinical patient characteristics and outcomes are presented in Table 3. Echocardiographic findings in *MYBPC3*^ΔInt32^ variant carriers ranged from normal to mild left atrial enlargement in one subject and severe HCM in another subject, indicating the importance of diagnostic testing to determine the presence of *MYBPC*^ΔInt32^ variant. However, a systematic study with more number of subjects is warranted to characterize the phenotypic outcome of the *MYBPC*^ΔInt32^ variant as it was involved in late onset and incomplete penetrance.

## Discussion

Cardiovascular disease accounted for 12.45 million out of over 56 million deaths worldwide in 2001, and it is a growing cause of mortality in South Asia [35]. South Asians originate from India, Pakistan, Sri Lanka, Nepal, Bangladesh, Afghanistan, and the Maldives. They represent a heterogeneous population, comprising a broad variety of diets, cultures, languages, religions, and lifestyles 36. Importantly, South Asians are at increased risk of developing coronary artery disease 20, 21, both in native and migrant populations, and they are also at a 3-to 5-fold increased risk for myocardial infarction and cardiovascular death compared to other ethnic groups [36–41].

With a diverse population of 1.671 billion (2013), preventative measures could be both cost-effective and substantial in decreasing the incidence of heart disease [41,42]. However, these preventative targets are still to be identified. HCM is most often characterized by unexplained left ventricular hypertrophy, but incomplete penetrance of HCM-causing mutations renders the disease unpredictable in terms of clinical outcome. Affected individuals may be asymptomatic or late-onset, developing symptoms in their third or fourth decade of life. Alternatively, patients with more extreme cases may present with arrhythmias, stroke, HF, and sudden cardiac death. Considering the high variability in HCM, genetic testing is crucial for risk assessment and proper clinical follow-up [43,44].

Currently, genetic testing consists of direct DNA sequencing for mutational analysis. To accomplish this, next-generation sequencing, massively parallel sequencing, and oligonucleotide hybridization chip-based technologies are valuable for high-throughput analysis of an individual’s complete library of disease-associated genes. As an alternative, individual clinical tests are available for HCM, dilated cardiomyopathy, and arrhythmogenic right ventricular cardiomyopathy and many specific known variants 45. However, such tests are fee-based, and the costs increase with complexity. Moreover, genetic testing on an individual basis makes population-wide disease analysis challenging [44].

In this study, we specifically developed a genotyping assay, termed RNaseH-based qPCR, for quick and affordable risk assessment of a sarcomeric protein mutation commonly found in South Asian individuals. This *MYBPC3*^ΔInt32^ variant has been previously established to cause various cardiomyopathies, including hypertrophic, dilated, and restrictive and coronary artery disease [20,21] in a sizeable percentage of South Asians [23]. Its presence was also found to be associated with development of left ventricular dysfunction in South Asian patients with coronary artery disease [20]. Heterozygous and homozygous carriers of this *MVBPC3*^ΔInt32^ have been shown to develop heart disease with adverse effects on quality of life and longevity. Based on the 2010 census, 11.22% of South Asians out of a total 3.1 million living in the U.S. have been found to carry the *MYBPC*^ΔInt32^ variant in *MYBPC3* [24]. This striking statistic supports the need for genotype/phenotype correlation and further genetic screening [24]. The assay we have presented could, therefore, become a sensitive, specific, and cost-effective method to determine the presence of this variant in South Asian individuals.

The development of this assay required several rounds of primer optimization as a result of difficulties with the nucleotide sequence created by the 25 bp deletion. Initial use of TaqMan-based PCR assays was unsuccessful, as the sequence flanking the 25 bp deletion produces an approximate 25 basepair sequence that would normally be specific for the mutant allele, but in this case, it also occurs verbatim in the WT sequence just downstream of the 25 bp deletion site (Figure 1b). This specific feature of the sequence may have contributed to the origin of the 25 bp deletion variant. Consequently, we needed to design a genotyping assay able to differentiate between full-length WT allele and the *MYBPC3*^ΔInt32^ variant. To do this, we first attempted using traditional splice site spanning primers, TaqMan approaches, locked nucleic acid (LNA) primers, and light upon extension (LUX) primers, all to no avail. Finally, we successfully designed a modified blocking strategy based on primer activation following RNaseH cleavage of a specific mismatched ribonucleotide, which drives preferential amplification of the desired sequence [45]. It is this blocking strategy that allows RNAseH primers to discriminate between WT and ΔInt32 alleles based on the first altered nucleotide 3’ to the 25 bp splice site [34,45]. The mechanics are as follows: 1) The forward primer contains the sequence that recognizes the template, followed by a single ribonucleotide base, followed by a non-extendable sequence (Figure 1b). 2) The forward WT or ΔInt32 forward primer anneals to the template. 3) If the single RNA base matches the target template, nothing happens. If the RNA base mismatches the template, then the RNaseH enzyme cleaves the RNA base. 4) Cleavage of the RNA base liberates the non-extendable 3’ blocking bases. 5) The primer is now able to extend and amplify the sequence.

This allows the assay to effectively discriminate templates based on single nucleotide changes, which can be problematic for other PCR-based methods. In this study, we have demonstrated an effective assay to determine the presence of the *MYBPC3*^ΔInt32^ variant in human genomic DNA isolated from both blood and saliva samples, targeting a South Asian cohort. We have shown that this assay has specificity for the *MYBPC3*^ΔInt32^ variant and that it can be used to blindly determine genotype based on differences in Cq values between the WT and ΔInt32 primers. This assay works efficiently and does not require the use of subsequent gel electrophoresis, allowing it to be used with ease in a clinical laboratory setting with minimal operator involvement. Furthermore, this approach is suitable for automation, allowing high-throughput, cost-effective analysis of hundreds of samples for a routine assay in a standard diagnostic laboratory without any advanced instrumentation. In addition, the high incidence of carriers of this mutation among South Asians living in the USA underscores the need for rapid diagnosis of those at high risk for development of cardiomyopathies [24].

With the successful implementation of the RNaseH qPCR assay, we will have established a foundational risk assessment screening technology that, by its single-step approach, will make automation very feasible, particularly in view of its potential widespread use. Screening combined with clinical evaluation will yield a wealth of genotype-phenotype data. Such data will help clarify whether *MYBPC3*^ΔInt32^ is a risk allele that works by exacerbating cardiovascular disease caused by other factors, either genetic or environmental, or if it is directly responsible for the development of cardiomyopathy. Indeed, our preliminary echocardiography findings, though limited by the small cohort, do show incomplete penetrance in *MYBPC*^ΔInt32^ variant carriers (Table 3). Accordingly, our future efforts will focus on performing a large-scale genotype coupled with clinical studies to accurately determine the role of this variant in the development of cardiovascular disease.

## Figures and Tables

**Table 1:** Blinded RNaseH genotyping results from 14 unknown human blood samples.

Genotype Call					
Sample ID	ΔCq	Observer 1	Observer 2	Observer 3	Confirmed Genotype
Unk B1	−5.43	WT	WT	WT	WT
Unk B2	0.16	Het	Het	Het	Het
Unk B3	−6.3	WT	WT	WT	WT
Unk B4	13.39	ΔInt32	ΔInt32	ΔInt32	ΔInt32
Unk B5	−6.83	WT	WT	WT	WT
Unk B6	11.88	ΔInt32	ΔInt32	ΔInt32	ΔInt32
Unk B7	−4.78	WT	WT	WT	WT
Unk B8	0.61	Het	Het	Het	Het
Unk B9	13.61	ΔInt32	ΔInt32	ΔInt32	ΔInt32
Unk B10	0.54	Het	Het	Het	Het
Unk B11	−7.45	WT	WT	WT	WT
Unk B12	−0.59	Het	Het	Het	Het
Unk B13	−0.53	Het	Het	Het	Het
Unk B14	0.53	Het	Het	Het	Het

Note: Three blinded observers were all in agreement with genotyping results by traditional PCR products run on an agarose gel. Criteria for genotyping were ΔCq values for unknown samples between −14 and −2 for WT, −2 and 2 for Het, and 8 and 19 for ΔInt32, as shown in Figures 4 and 5.

**Table 2:** Blinded RNaseH genotyping results from 12 unknown human saliva samples.

Genotype Call					
Sample ID	ΔCq	Observer 1	Observer 2	Observer 3	Confirmed Genotype
Unk S1	−3.25	WT	WT	WT	WT
Unk S2	1.05	Het	Het	Het	Het
link S3	−7.58	WT	WT	WT	WT
Unk S4	1.96	Het	Het	Het	Het
Unk S5	−7.66	WT	WT	WT	WT
Unk S6	−9.22	WT	WT	WT	WT
Unk S7	−7.75	WT	WT	WT	WT
Unk S8	−7.12	WT	WT	WT	WT
Unk S9	−3.61	WT	WT	WT	WT
UnkS10	−9.53	WT	WT	WT	WT
UnkS11	−9.22	WT	WT	WT	WT
UnkS12	−6.79	WT	WT	WT	WT

Note: Three blinded observers were all in agreement with genotyping results by traditional PCR products run on an agarose gel. Criteria for genotyping were ΔCq values for unknown samples between −14 and −2 for WT, −2 and 2 for Het, and 8 and 19 for ΔInt32, as shown in Figures 4 and 5.

**Table 3:** Echocardiography results from 9 heterozygous *MYBPC3*^ΔInt32^ variant carriers.

Ref	Geno type	Age (Yrs)	Gender	BP (mmHg)	Fs (%)	EF (%)	LVDD (cm)	LVSD (cm)	IVST (cm)	LITIVE (cm)	E/A ratio	E/E’ ratio	Conclusions In Clinical Report
1	Carrier	38	M	109/60	32.5	60.7	4.8	3.2	0.84	0.8	1.7	9.2	Normal global left ventricular ejection fraction.
2	Carrier	42	M	124/74	37.5	59.2	4.3	2.7	1.2	0.83	1.3	9.2	Normal global left ventricular ejection fraction. Normal echocardiogram.
3	Carrier	43	F	Not Found	34	63.4	4.1	2.7	0.39	0.89	1.4	12.3	Normal global left ventricular ejection fraction. Normal echocardiogram
4	Carrier	43	F	110/60	25.3	50.2	4.3	3.2	0.7	0.8	2.4	8.5	Normal global left ventricular ejection fraction.
5	Carrier	45	M	133/79	29.7	57.3	4.1	2.9	1.6	1.5	1.9	27.2	Severe left ventricular hypertrophy; hypertrophic cardiomyopathy; left ventricualar diastolic dysfunction; mild left atrial enlargement.
6	Carrier	45	M	145/86	37.4	68.1	3.9	2.5	1	0.92	1.1	10.7	Normal global left ejection fraction.
7	Carrier	47	M	107/65	39	69.8	4.3	2.6	0.78	0.68	1.5	8.5	Mildly dilated aortic root. Normal global left ventricular ejection fraction.
8	Carrier	54	M	125/72	35.1	64.1	5	3.3	0.91	0.71	1.7	10.3	Normal global left ventricular ejection fraction.
9	Carrier	61	M	122/70	37	66.6	5.1	3.2	0.82	0.59	0.96	6.4	Normal global left ventricular ejection fraction. Mild left atrial enlargement. Mildly abnormal echocardiogram

Note: BP=Blood Pressure; EF=Ejection Fraction; FS= Fractional Shortening; IVST=Interventricular Septal Thickness; LVDD=Left Ventricular Diastolic Diameter; LVSD=Left Ventricular Systolic Diameter; LVPWT=Left Ventricular Posterior Wall Thickness.

According to the European Society for Cardiology [5], HCM is defined in adults by an IVST greater than 1.5 cm in one or more LV myocardial segments. Based on these definitions, subject # 5 is definite HCM, with IVST of 1.6 cm and LVPWT of 1.5 cm. In addition, two other subjects, # 7 and # 9 had mild arterial abnormalities.

## Abbreviations

cMyBP-C: Cardiac Myosin Binding Protein-C
*MYBPC3*: Cardiac Myosin Binding Protein-C Gene
*MYBPC3*^ΔInt32^: Intron 32 Mutant Cardiac Myosin Binding Protein-C
HCM: Hypertrophic Cardiomyopathy
cMyBP-Cwt: wild-type cMyBP-C
Qpcr: Quantitative Real-Time Polymerase Chain Reaction
UTR: Untranslated Region
Cq: Cycle of Quantitation
